# Toward Point-of-Care Detection of *Mycobacterium tuberculosis*: A Brighter Solvatochromic Probe Detects Mycobacteria within Minutes

**DOI:** 10.1021/jacsau.1c00173

**Published:** 2021-07-26

**Authors:** Mireille Kamariza, Samantha G. L. Keyser, Ashley Utz, Benjamin D. Knapp, Christopher Ealand, Green Ahn, C. J. Cambier, Teresia Chen, Bavesh Kana, Kerwyn Casey Huang, Carolyn R. Bertozzi

**Affiliations:** † Department of Biology, Stanford University, Stanford, California 94305, United States; ‡ Department of Chemistry, University of California, Berkeley, Berkeley, California 94720, United States; § Biophysics Program, Stanford University, Stanford, California 94305, United States; ∥ Department of Science and Technology/National Research Foundation Centre of Excellence for Biomedical Tuberculosis Research, Faculty of Health Sciences, University of Witwatersrand, National Health Laboratory Service, Johannesburg 2000, South Africa; ⊥ Department of Chemistry, Stanford University, Stanford, California 94305, United States; # Medical Research Council−Centre for the AIDS Programme of Research in South Africa (CAPRISA) HIV-TB Pathogenesis and Treatment Research Unit, Durban 4013, South Africa; ∇ Department of Bioengineering, Stanford University, Stanford, California 94305, United States; ○ Department of Microbiology and Immunology, Stanford University School of Medicine, Stanford, California 94305, United States; ◆ Chan Zuckerberg Biohub, San Francisco, California 94158, United States; ¶ Howard Hughes Medical Institute, Stanford University, Stanford, California 94305, United States

**Keywords:** *Mycobacterium tuberculosis*, solvatochromism, environment-sensitive dyes, fluorescence, trehalose metabolism, point-of-care TB detection

## Abstract

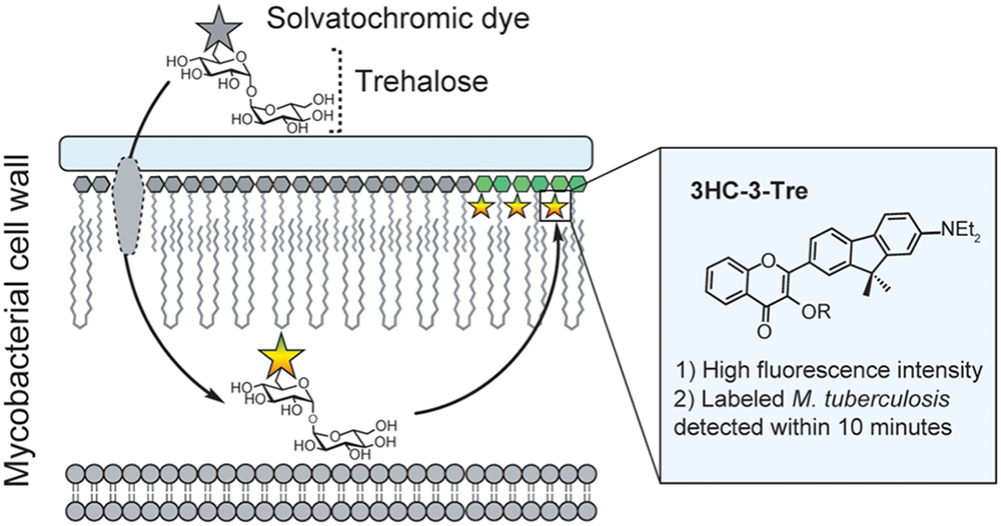

There is an urgent need for point-of-care tuberculosis (TB) diagnostic methods that are fast, inexpensive, and operationally simple. Here, we report on a bright solvatochromic dye trehalose conjugate that specifically detects *Mycobacterium tuberculosis* (Mtb) in minutes. 3-Hydroxychromone (3HC) dyes, known for having high fluorescence quantum yields, exhibit shifts in fluorescence intensity in response to changes in environmental polarity. We synthesized two analogs of 3HC-trehalose conjugates (3HC-2-Tre and 3HC-3-Tre) and determined that 3HC-3-Tre has exceptionally favorable properties for Mtb detection. 3HC-3-Tre-labeled mycobacterial cells displayed a 10-fold increase in fluorescence intensity compared to our previous reports on the dye 4,4-*N,N*-dimethylaminonapthalimide (DMN-Tre). Excitingly, we detected fluorescent Mtb cells within 10 min of probe treatment. Thus, 3HC-3-Tre permits rapid visualization of mycobacteria that ultimately could empower improved Mtb detection at the point-of-care in low-resource settings.

## Introduction

With 1.2 million deaths and 10 million new cases in 2018, tuberculosis (TB) is the most lethal infectious disease in the world.^1^ Early detection of the bacterium *Mycobacterium tuberculosis* (Mtb), the causative agent of TB, followed by appropriate treatment could prevent most deaths.^2^ The gold standard for TB diagnosis remains a labor-intensive culture test that requires weeks of incubation time in specialized facilities. Although more rapid tests are available, they present several important limitations. PCR-based tests are expensive and require skilled technicians. Microscopy-based methods are attractive in low-resource settings as they are low-cost, have fast turnaround times and report on people at greatest risk of transmission and death.^3^ As a result, the sputum smear microscopy test is the most widely used technique for TB diagnosis. The century-old smear test is based on the propensity of fluorescent auramine dye or colored Ziehl-Neelson (ZN) stain to accumulate within the highly hydrophobic mycobacterial cell wall.^4−7^ While effective for identification of Mtb cells, this process requires multiple wash steps to reduce nonspecific background fluorescence, or in the case of the ZN test, a rigorous counterstaining procedure so that stained Mtb cells can be visualized.^8,9^ Moreover, the smear test does not distinguish live from dead cells, and this capability is vital in order to assess treatment efficacy early and accurately.^2^

In the past decade, we and others have leveraged the trehalose metabolism of mycobacteria to mark them for detection by various imaging methods.^10−20^ Exogenous trehalose molecules can be directly mycolylated at the 6 position by antigen 85 (Ag85) enzymes to form trehalose monomycolates (TMM) that are inserted into the mycobacterial cell wall, termed the mycomembrane.^10^ Researchers have shown that Ag85 enzymes are promiscuous enough to tolerate perturbations of varying sizes such as azide,^11^ alkyne,^12^ fluorine,^13−15^ and fluorophore^13,16^ groups that permit visualization of the mycomembrane as long as the cell is metabolically active. However, these fluorescent probes require extensive washing before imaging in order to reduce background signal.

**Figure 1:**
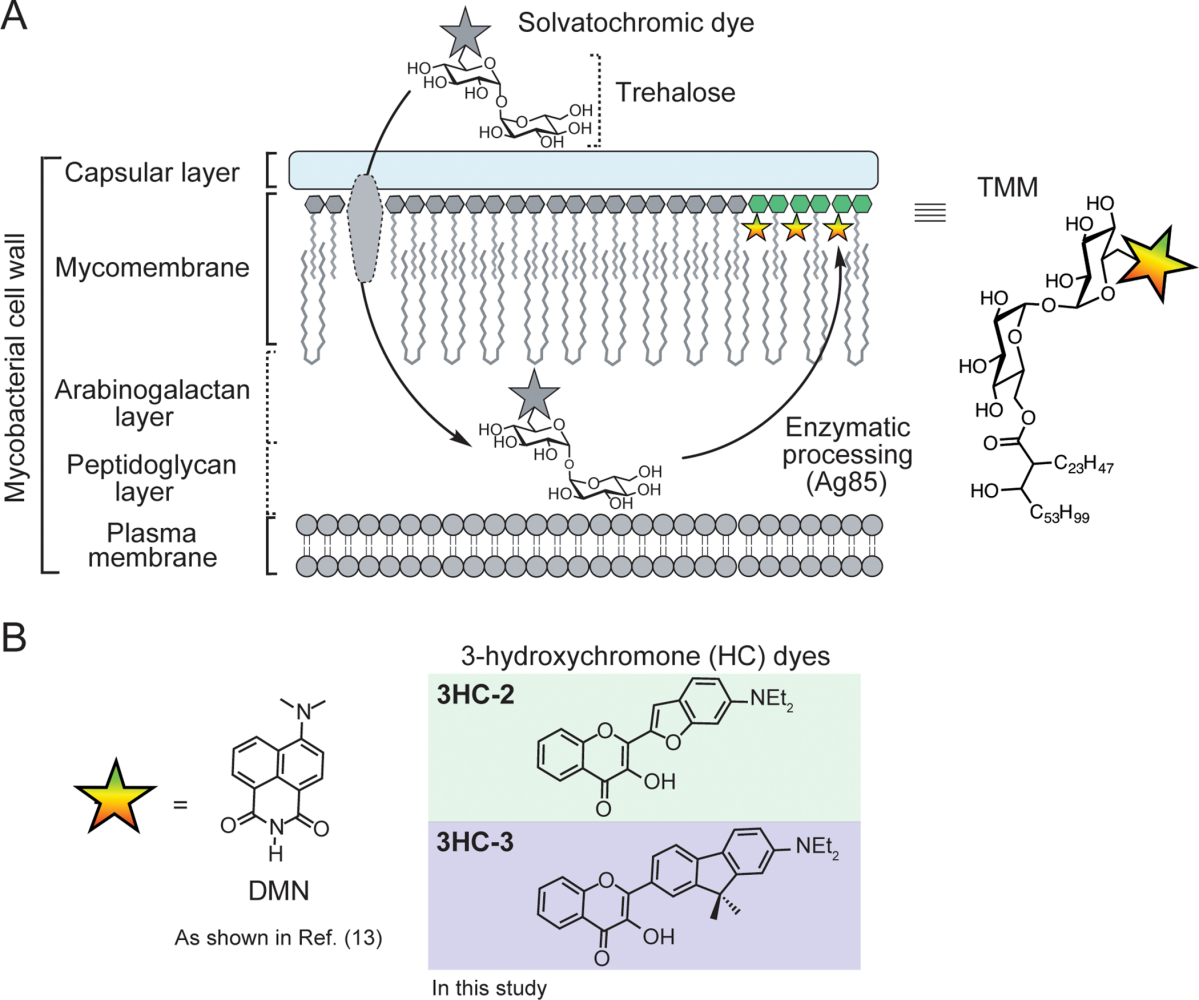
Solvatochromic trehalose probes label the mycobacterial mycomembrane. (A) Solvatochromic trehalose probes are converted by mycobacteria to the corresponding trehalose monomycolate (TMM, structure on right) analogs and inserted into the mycomembrane. There, they undergo fluorescence turn-on, enabling detection of labeled cells by fluorescence microscopy. (B) Chemical structures of solvatochromic dyes described in this study.

Fluorogenic probes, that is, probes that turn on when metabolized in cells, have proven better suited for TB detection as they require minimal processing. Previous studies have used quenched trehalose fluorophores that become unquenched by Ag85 activity, allowing visualization of growing mycobacterial cells in real-time.^17^ A dual enzyme-targeting fluorogenic probe allowed the detection of Mtb cells within an hour using a microfluidic system.^18^ In addition, we reported on a solvatochromic trehalose probe (DMN-Tre) that can detect live Mtb cells in TB patient sputum samples.^19^ Solvatochromic dyes change their color or fluorescence intensity based on the polarity of the solvent. As a result, these compounds are advantageous for monitoring changes in hydrophobicity around a molecule of interest.^20^ Upon acylation of DMN-Tre by Ag85 and insertion of the corresponding trehalose monomycolate (TMM) analog into the mycomembrane, dye fluorescence is turned on, which allows the detection of live mycobacteria with a fluorescence microscope (Figure 1A).^19,21^ In this context, DMN-Tre has many favorable properties for point-of-care deployment: its operationally simple procedure does not require any wash steps, and it is synthetically convenient and chemically stable. However, the DMN dye is a fluorophore of relatively low brightness with a molar extinction coefficient of 8800 M^–1^ cm^–1^ in TBS buffer^20^ and low quantum yield of fluorescence in low-dielectric solvents (0.288 in DMF).^22^ Consequently, in practice we found that low-powered fluorescence microscopes currently available in TB health centers may miss labeled cells and that Mtb cells must be labeled with DMN-Tre for at least 1 h to achieve detectable levels of fluorescence using standard clinical fluorescence microscopes.

To address these obstacles for point-of-care TB detection, we aimed to develop probes that are brighter and enable faster detection of live Mtb cells. Here, we report the development of a brighter solvatochromic trehalose probe, based on the 3-hydroxychromone (3HC) dye (Figure 1B). The 3HC trehalose conjugate (termed 3HC-3-Tre) demonstrated high fluorescence turn-on in hydrophobic solvents as well as when incorporated in the mycobacterial cell surface. This labeling was specific to the trehalose moiety and was detectable without any wash steps. Additionally, the fluorescence intensity of 3HC-3-Tre was 10-fold brighter than DMN-Tre. Finally, the high signal-to-noise ratio of 3HC-3-Tre permitted simple detection of labeled Mtb cells within 10 min. Thus, 3HC-3-Tre reagent permits rapid visualization of mycobacteria and ultimately could be used to improve Mtb detection in low-resource environments.

## Results

### Synthesis of 3HC Solvatochromic Dyes Bound to Trehalose

To design a probe with stronger turn-on fluorescence than DMN, we relied on previously reported solvatochromic dyes that fit our target profile. We identified a highly promising class of solvatochromic dyes that are well characterized, synthetically tractable, and showed minimal perturbations in living systems (Figure 1B).^20^ These dyes are based on a 3-hydroxychromone (3HC) scaffold with the advantage of greater tunability due to their synthetic modularity and high quantum fluorescence yield. Moreover, they are further red-shifted, which may minimize background fluorescence.

**Figure 2:**
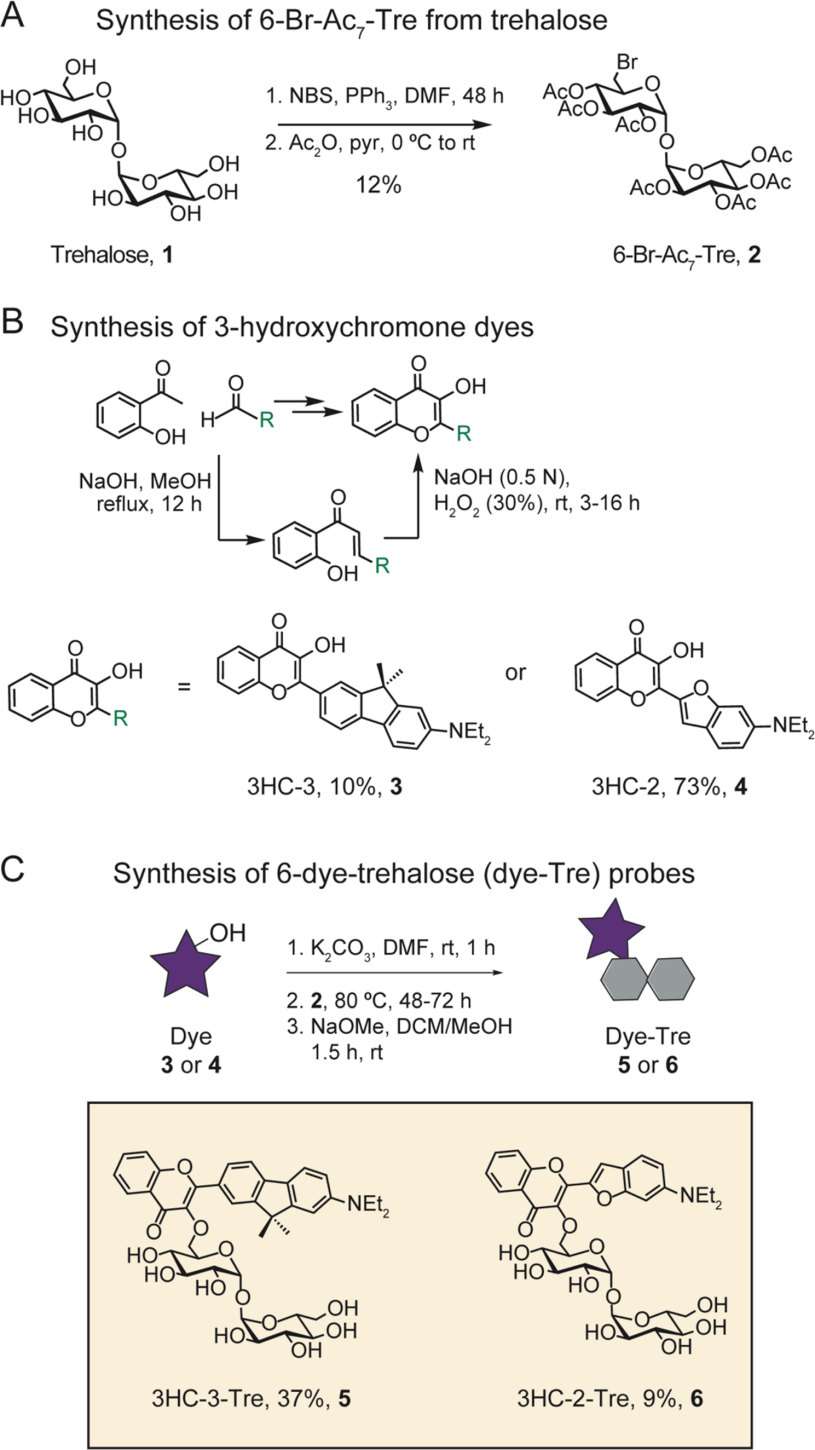
Synthesis scheme for 3-hydroxychromone (3HC) trehalose (Tre) dye conjugates.

We began by synthesizing 6–Br-Ac_7_-Tre (Figure 2). An Appel reaction using N-bromosuccinimide and triphenylphosphine resulted in a mixture of monobrominated target compound (6-Br-Tre), a dibrominated side product (6,6′-dibromo-6,6′-dideoxy-α,α′-trehalose) and unreacted starting material. The crude material was then acetylated and purified to give 6-Br-Ac_7_-Tre (Compound 2). Next, we considered the synthesis of 3HC dyes, here referred to as 3HC-3 (Compound 3) and 3HC-2 (Compound 4).^23−26^ We followed a previous study^23^ to achieve synthesis of the aldehyde precursor to 3HC-2 by reacting 3-diethylaminophenol with bromoacetaldehyde diethyl acetal (Scheme S1). The intermediate was purified, then subsequently treated with phosphorus (V) oxychloride and *N*,*N*-dimethylformamide (DMF) to form the benzofuran and to install an aldehyde at the 2-position via a Vilsmeier–Haack reaction. To synthesize the aldehyde precursor to 3HC-3 (Compound 3), we nitrated 2-bromo-9,9-dimethylfluorene at the 7-position, reduced the nitrate to an amine, alkylated the amine using ethyl iodide and converted the bromine to an aldehyde through a Bouveault reaction. To form 3HC-2 and 3HC-3, each aldehyde was reacted with 2′-hydroxyacetophenone, then treated with hydrogen peroxide to obtain the desired products (Figure 2B).^27^ Finally, to create the dye-Tre probes, the aromatic hydroxyl group on the dye was deprotonated with potassium carbonate and used to displace the bromine atom on 6-Br-Ac_7_-Tre (Figure 2C).^28^ The crude dye-Ac_7_-Tre was then deacetylated with catalytic sodium methoxide and purified to produce the final dye-Tre products in yields ranging from 9 to 63% over three steps. We also synthesized glucose control compounds in a similar fashion (Scheme S2).

### 3HC-Trehalose Probes Label *Mycobacterium smegmatis* (Msmeg) Cells More Effectively than DMN-Tre

**Figure 3:**
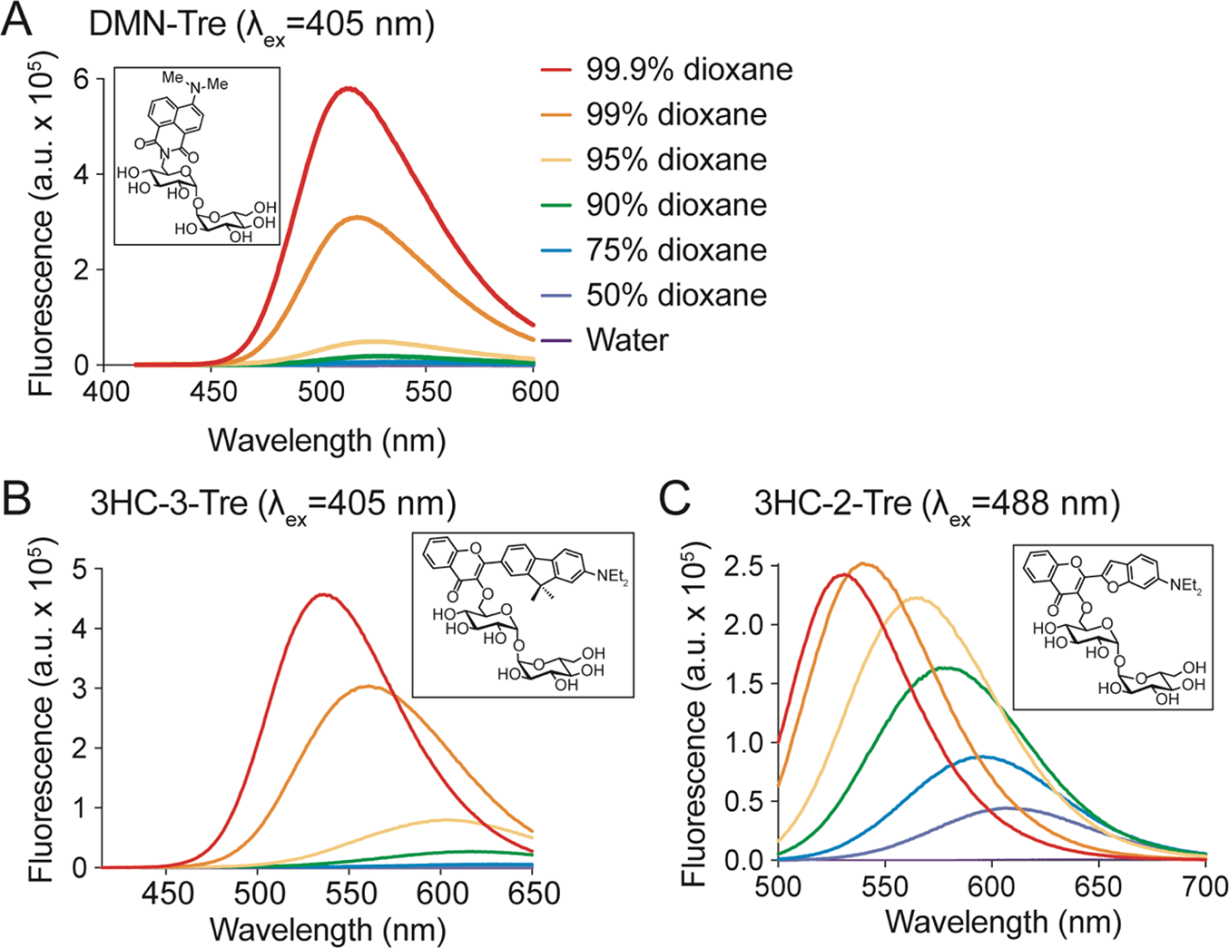
Emission spectra of 3-hydroxychromone trehalose dyes. Fluorescence spectra of (A) DMN-Tre (ex. 405 nm), (B) 3HC-3-Tre (ex. 405 nm), and (C) 3-HC-2-Tre (ex. 488 nm) in solvent systems with the indicated ratios of dioxane in water.

With these probes in hand, we proceeded to characterize their fluorescence intensities and emission spectra in mixtures with various ratios of dioxane and water. The dyes were excited at the optimal excitation wavelengths (405 nm for DMN-Tre and 3HC-3-Tre, 488 nm for 3HC-2-Tre) and fluorescence intensities were measured over a range of emission wavelengths (Figure 3). As expected, all probes displayed increased fluorescence as the amount of dioxane increased. DMN-Tre and 3HC-3-Tre both absorbed most strongly at 405 nm and had comparable fluorescence intensities in each solvent mixture tested, although the spectra for 3HC-3-Tre were slightly red-shifted (Figure 3A,B). 3HC-2-Tre responded well to excitation at 405 and 488 nm, although its fluorescence intensity was less sensitive to changes in hydrophobicity overall (Figure 3C). However, because the emission spectra underwent a bathochromic shift as solvent polarity increased, significant differences in intensity still occurred between 500 and 550 nm, the approximate range of wavelengths allowed through the GFP emission filter.

**Figure 4:**
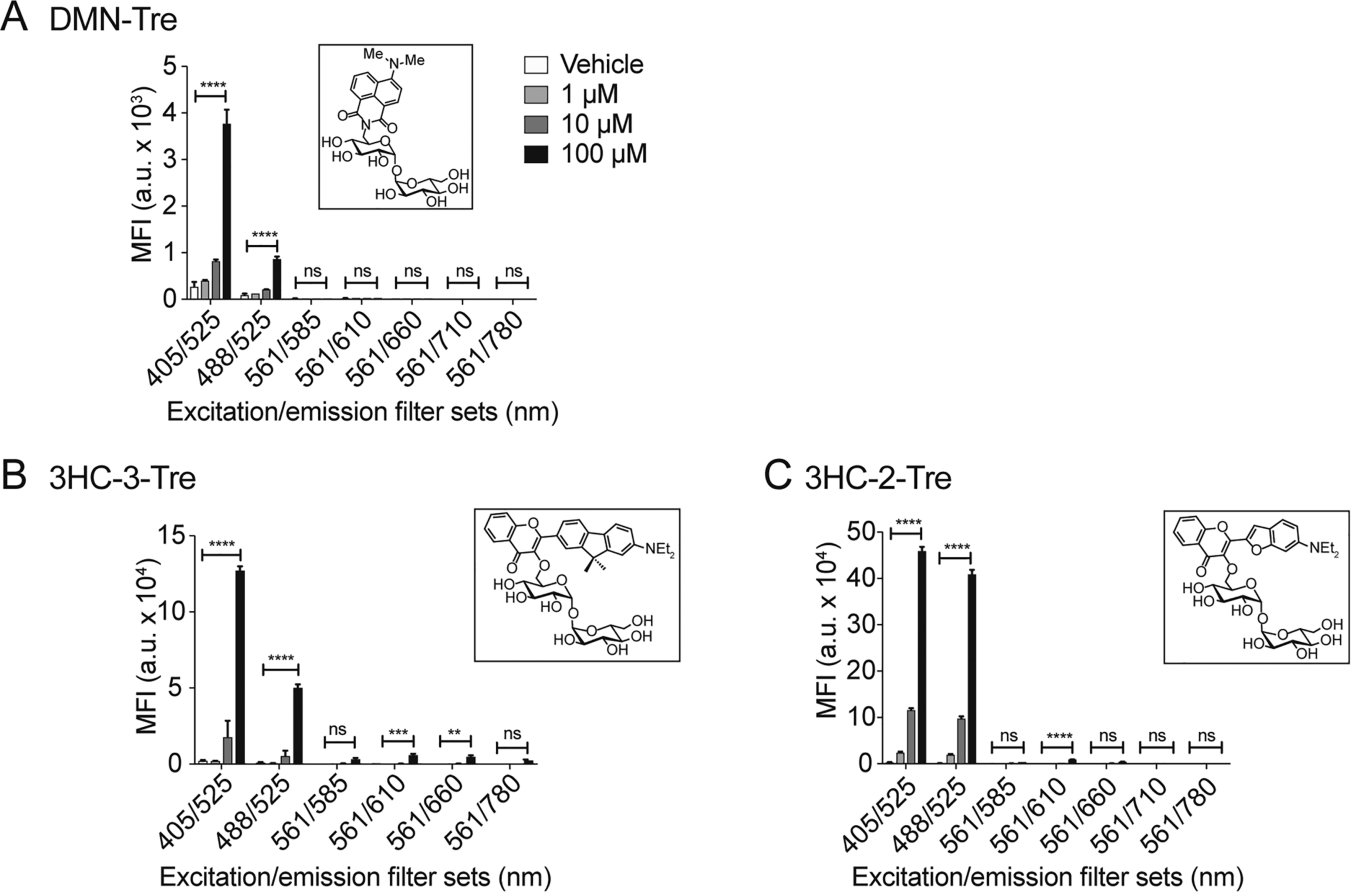
Flow cytometry analysis of Msmeg cells labeled with solvatochromic trehalose dyes using various excitation and emission filter sets. Flow cytometry analysis of Msmeg labeled with (A) DMN-Tre, (B) 3HC-3-Tre, or (C) 3HC-2-Tre. All dyes showed increased labeling at the highest concentration (100 μM). Cells at OD_600_ = 0.5 were incubated with the indicated dye-trehalose probe concentrations for 1 h at 37 °C. MFI, mean fluorescence intensity. Data are means ± SEM from at least two independent experiments. Data were analyzed by two-way ANOVA tests for unequal variances with Dunn’s multiple comparisons test using selected adjusted *P* values. (*, *p* < 0.05; **, *p* < 0.01; ***, *p* < 0.001; ****, *p* < 0.0001; ns, not significant).

We also assessed labeling conditions for *Mycobacterium smegmatis* (Msmeg), a nonpathogenic and fast-growing member of the *Mycobacterium* genus commonly used as a model organism for Mtb. Msmeg cells were grown to an optical density at wavelength 600 nm (OD_600_) of 0.5, then incubated with 1, 10, or 100 μM of each probe for 1 h at 37 °C, washed three times, and analyzed by flow cytometry using a variety of excitation and emission filter sets (Figure 4). As expected, we observed that all trehalose probes labeled Msmeg in a concentration-dependent manner. Moreover, 3HC-3-Tre and 3HC-2-Tre-labeled cells reached much higher levels of fluorescence intensity compared to DMN-Tre-labeled bacteria (approximately 10-fold and 100-fold higher for 3HC-3-Tre and 3HC-2-Tre, respectively). While DMN-Tre’s fluorescence was optimally detected with 405/525 (ex/em) nm filter sets (Figure 4A), we determined that the optimal fluorescence detection filter sets for 3HC-3-Tre and 3HC-2-Tre probes were 405/525 and 488/525 nm, respectively (Figure 4B,C). Excitingly, even with 10-fold lower concentrations, 3HC-3-Tre-labeled Msmeg cells demonstrated nearly 5-fold greater fluorescence intensity compared to DMN-Tre.

### 3HC-3-Tre Rapidly and Stably Labels Mycobacteria in a Trehalose-Dependent Manner

**Figure 5:**
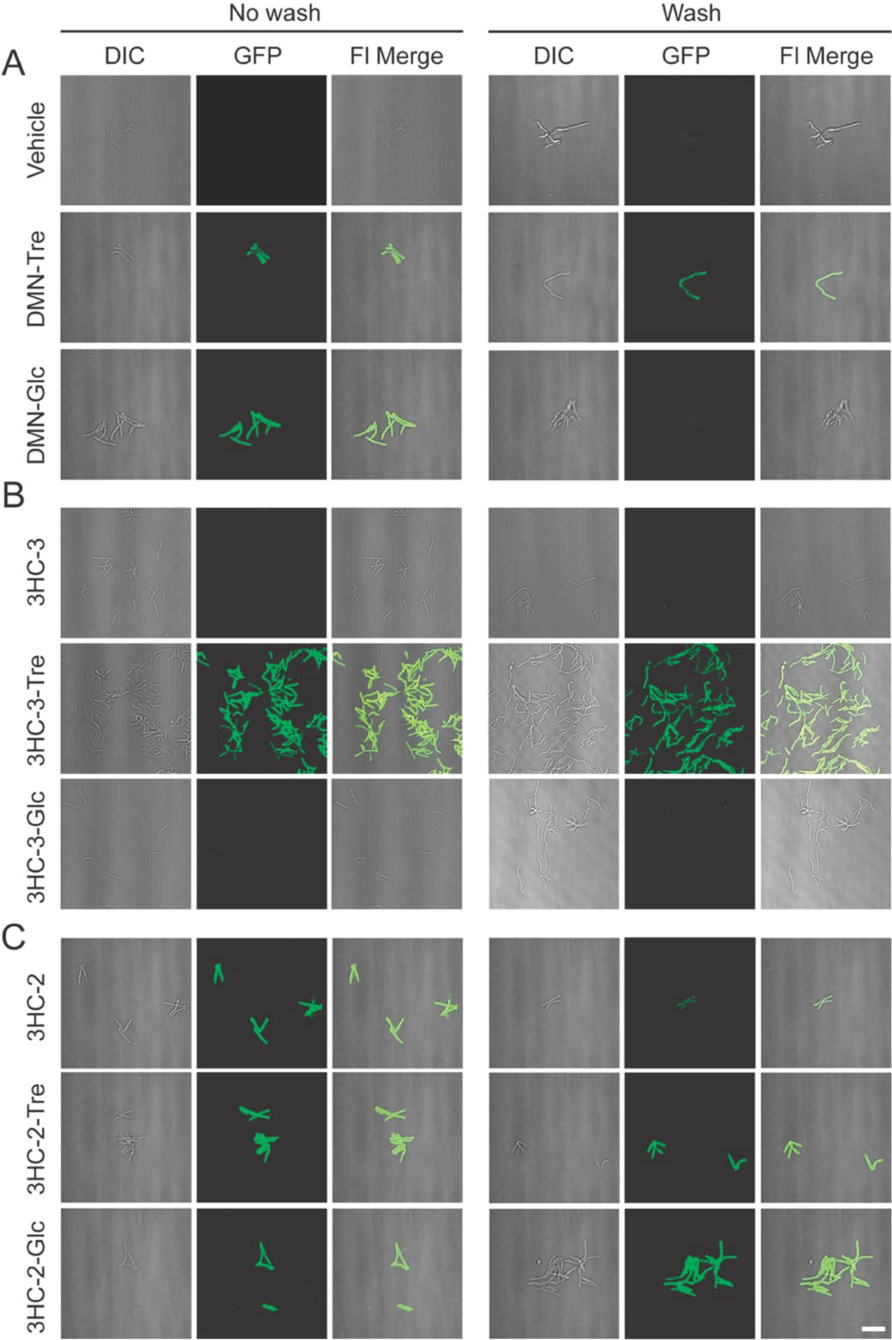
Unlike 3HC-2-Tre, 3HC-3-Tre labeling is dependent on the trehalose moeity. Epifluorescence microscopy of Msmeg cells treated with (A) 100 μM DMN-Tre or DMN-Glc or no dye control (Vehicle); (B) 100 μM of 3HC-3, 3HC-3-Tre, or 3HC-3-Glc; (C) 100 μM of 3HC-2, 3HC-2-Tre, or 3HC-2-Glc. 3HC-3-Tre showed the most efficient labeling of Msmeg. Cells were incubated with the indicated dyes for 1 h at 37 °C. Cells were smeared directly (No wash) or washed 3 times with PBS then smeared onto a microscope slide (Wash). Scale bar: 10 μm.

For dyes to be useful in the field, the labeling procedure must follow a simple protocol. Thus, we sought to determine whether a wash step is necessary based on the degree of background signal when Msmeg cells are labeled with each trehalose probe, glucose-dye control, or free dye. We incubated Msmeg cells with DMN-Tre, 3HC-3-Tre, or 3HC-2-Tre (along with their respective glucose and sugar-free analogs) for 1 h at 37 °C and performed fluorescence microscopy either immediately or after a wash step with PBS (Figure 5). As anticipated, we observed fluorescent Msmeg cells labeled with DMN-Tre, while the fluorescence of DMN-Glc-labeled Msmeg was largely eliminated with PBS (Figure 5A), suggesting that the nonspecific turn-on of DMN-Glc molecules is likely due to proximity to Msmeg cells. Surprisingly, we observed no fluorescence with 3HC-3-Glc-labeled Msmeg cells, even in unwashed samples (Figure 5B, Figure S1A), suggesting that internalization of the probe is required for fluorescence turn-on of 3HC-3. Interestingly, we observed labeling of Msmeg cells with 3HC-2 and 3HC-2-Glc, even after washing (Figure 5C, Figure S1B), suggesting that 3HC-2 labeling is not specific to the trehalose pathway. Intrigued by this result, we wondered whether the fluorescence labeling of Msmeg from 3HC-2 dye conjugates would reflect the known trehalose insertion patterning.

news

**Figure 6:**
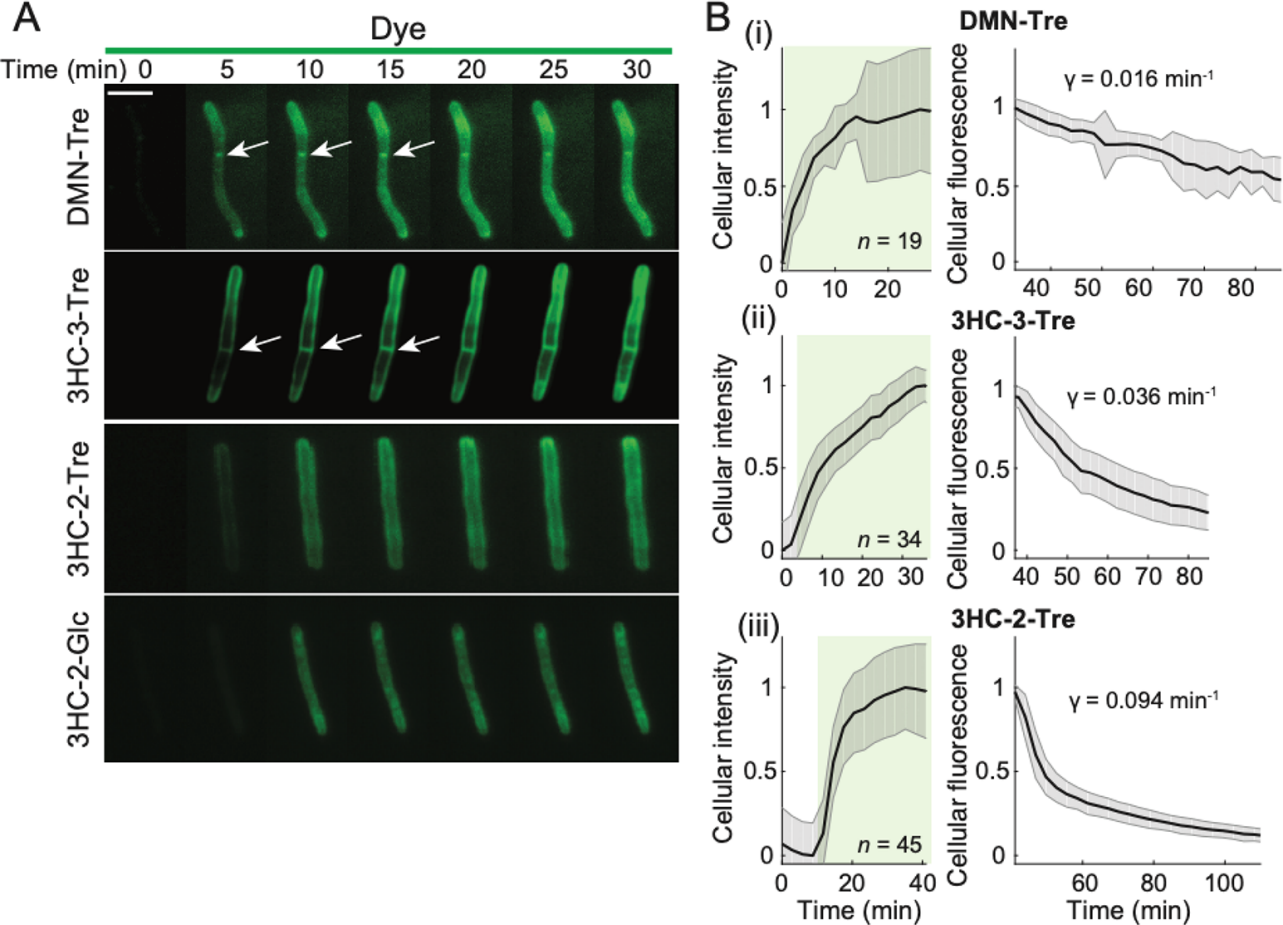
Unlike 3HC-2 dye conjugates, 3HC-3-Tre labeling is initially localized at the septum and poles. (A) Time-lapse microscopy of Msmeg cells treated with 100 μM DMN-Tre, 3HC-3-Tre, 3HC-2-Tre, or 3HC-2-Glc for 30 min revealed concentration of 3HC-3-Tre at cell septa and poles. White arrows denote septal labeling. Scale bar: 5 μm. (B) Quantification of Msmeg fluorescence in the presence of 100 μM (i) DMN-Tre, (ii) 3HC-3-Tre, or (iii) 3HC-2-Tre during labeling for 30 min (left, volume-normalized intensity) and subsequent washing with growth medium for 1 h (right, total fluorescence). The number of cells included in each analysis (*n*) is provided in each panel. Shaded error bars represent ±1 standard deviation.

Previous studies demonstrated that exogenous trehalose molecules are mycolylated at the 6 position via action of Ag85 enzymes, which are localized at the septa and poles of the cell envelope.^10,19^ We hypothesized that 3HC-2 labeling occurs in an Ag85-independent manner and therefore would not exhibit polar and septal fluorescence. Using total internal reflection fluorescence (TIRF) microscopy, we placed Msmeg cells into a microfluidic flow cell (Methods), introduced liquid growth medium containing DMN-Tre, 3HC-3-Tre, 3HC-2-Tre, or 3HC-2-Glc, and performed time-lapse microscopy (Figure 6, Figure S2). Similar to DMN-Tre, 3HC-3-Tre labeling of Msmeg cells showed septal and polar labeling (Figure 6A, top two panels). We observed no enhancement in polar or septal fluorescence localization for Msmeg cells labeled with 3HC-2-Tre or 3HC-2-Glc (Figure 6A, bottom two panels), further suggesting that this labeling likely does not depend on the trehalose metabolic pathway.

After 30 min of labeling, we washed out the exogenous dye and continued to acquire fluorescence images. To address the rate of fluorescence change during labeling and washout, we extracted the total fluorescence within each cell at each time point and quantified the mean total and volume-normalized fluorescence across cells (Figure 6B). During labeling, the volume-normalized fluorescence initially increased rapidly and then started to plateau; by 10 min, cells reached ∼50% of the labeling at 30 min, confirming rapid labeling of all three probes. During washout, total fluorescence was gradually lost (Figure 6B). We fit the washout dynamics to an exponential (*t*) = *I*_0_ + *I*_1_*e*^–*γt*^. Washout of DMN-Tre labeling was slow, likely because it depends on mycomembrane turnover (Figure 6Bi, Figure S2A). Compared to DMN-Tre, cells labeled with 3HC-3-Tre or 3HC-2-Tre exhibited a rate of fluorescence loss γ that was 2.3- and 5.9-fold higher than DMN-Tre, respectively (Figure 6Bii,iii, Figure S2B,C), suggesting additional labeling mechanisms beyond trehalose synthesis. In particular, the washout time scale for 3HC-2-Tre was ln 2/γ = 7.4 min, reflecting trehalose-independent transient binding and/or turn-on. Nonetheless, we confirmed that 3HC-3 and 3HC-3-Glc do not label Msmeg cells (Figure S2E,F), indicating that trehalose is necessary for successful labeling of Msmeg cells. Taken together, these data demonstrate that 3HC-3-Tre, but not 3HC-2-Tre, is an excellent candidate for the rapid detection of mycobacteria. Moving forward, we focused our efforts on the 3HC-3-Tre probe.

### 3HC-3-Tre Labeling of Msmeg Cells Is Much Brighter than DMN-Tre and Selective for Actinobacteria

**Figure 7:**
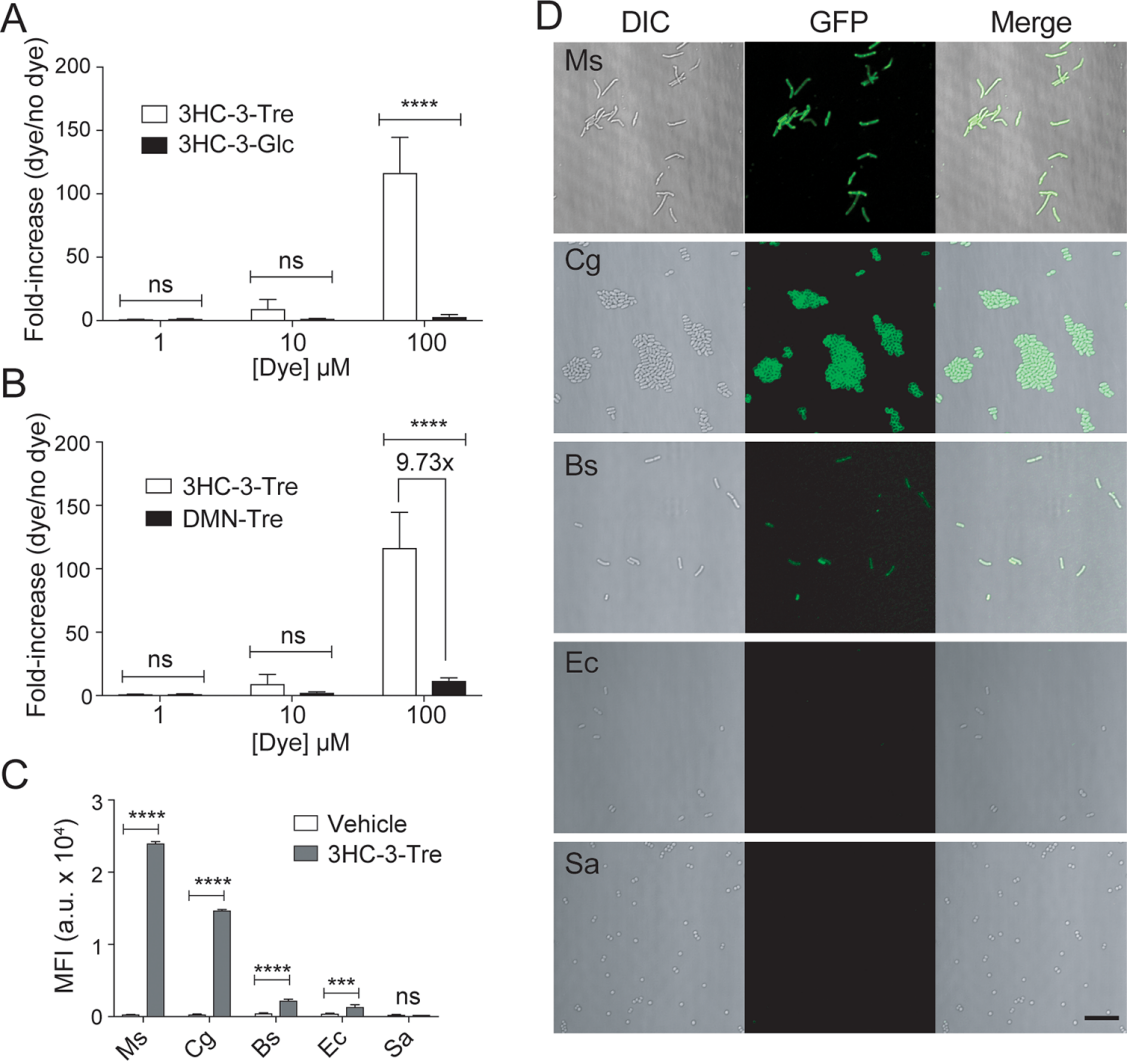
Specific labeling of live mycobacteria and corynebacteria with 3HC-3-Tre. (A,B) Flow cytometry analysis of Msmeg cells incubated for 1 h at 37 °C with (A) 100 μM 3HC-3-Tre or 3HC-3-Glc, (B) 100 μM 3HC-3-Tre or DMN-Tre. (C,D) Flow cytometry (C) and no-wash microscopy (D) analyses of Msmeg (Ms), *C. glutamicum* (Cg), *B. subtilis* (Bs), *E. coli* (Ec), and *S. aureus* (Sa) cells incubated for 1 h at 37 °C with 100 μM 3HC-3-Tre. 3HC-3-Tre labeling was specific to Msmeg and Cg. Data are means ± SEM from at least two independent experiments. Data were analyzed by two-way ANOVA tests for unequal variances with Dunn’s multiple comparisons test using selected adjusted *p*-values (***, *p* < 0.001; ****, *p* < 0.0001; ns, not significant). Scale bar: 10 μm.

Our next goal was to assess the specificity of 3HC-3-Tre labeling of mycobacteria compared with bacterial species that do not incorporate trehalose into their cell envelopes (Figure 7). We analyzed Msmeg cells incubated with 1, 10, or 100 μM of 3HC-3-Tre or 3HC-3-Glc (as a negative control) for 1 h at 37 °C. We found that 100 μM 3HC-3-Tre-labeled cells were 100-fold brighter compared to background (Figure 7A). Importantly, in the same conditions, the fluorescence intensity of 3HC-3-Tre-labeled cells was 10-fold greater than that of DMN-Tre-labeled cells (Figure 7B), confirming that 3HC-3-Tre is a much brighter option than DMN-Tre. We previously reported that DMN-Tre selectively labeled organisms within the Actinobacteria suborder.^16,19^ To test whether a similar labeling pattern persisted for 3HC-3-Tre, we labeled Msmeg, *Corynebacterium glutamicum* (Cg), *Bacillus subtilis* (Bs), *Escherichia coli* (Ec), and *Staphylococcus aureus* (Sa) with 100 μM 3HC-3-Tre for 1 h and analyzed the samples by microscopy and flow cytometry (Figure 7C,D). Similar to DMN-Tre, we observed bright labeling of Msmeg and Cg cells, consistent with both of these organisms utilizing trehalose in their cell envelopes. There was minimal but significant fluorescence from labeled Bs, Ec, and Sa cells compared to the no-dye control using flow cytometry (Figure 7C), perhaps reflecting a trehalose-independent pathway that led to faster washout of 3HC-3-Tre than DMN-Tre (Figure 6B).

### 3HC-3-Tre Labeling of Mtb Is Detectable within 10 min

**Figure 8:**
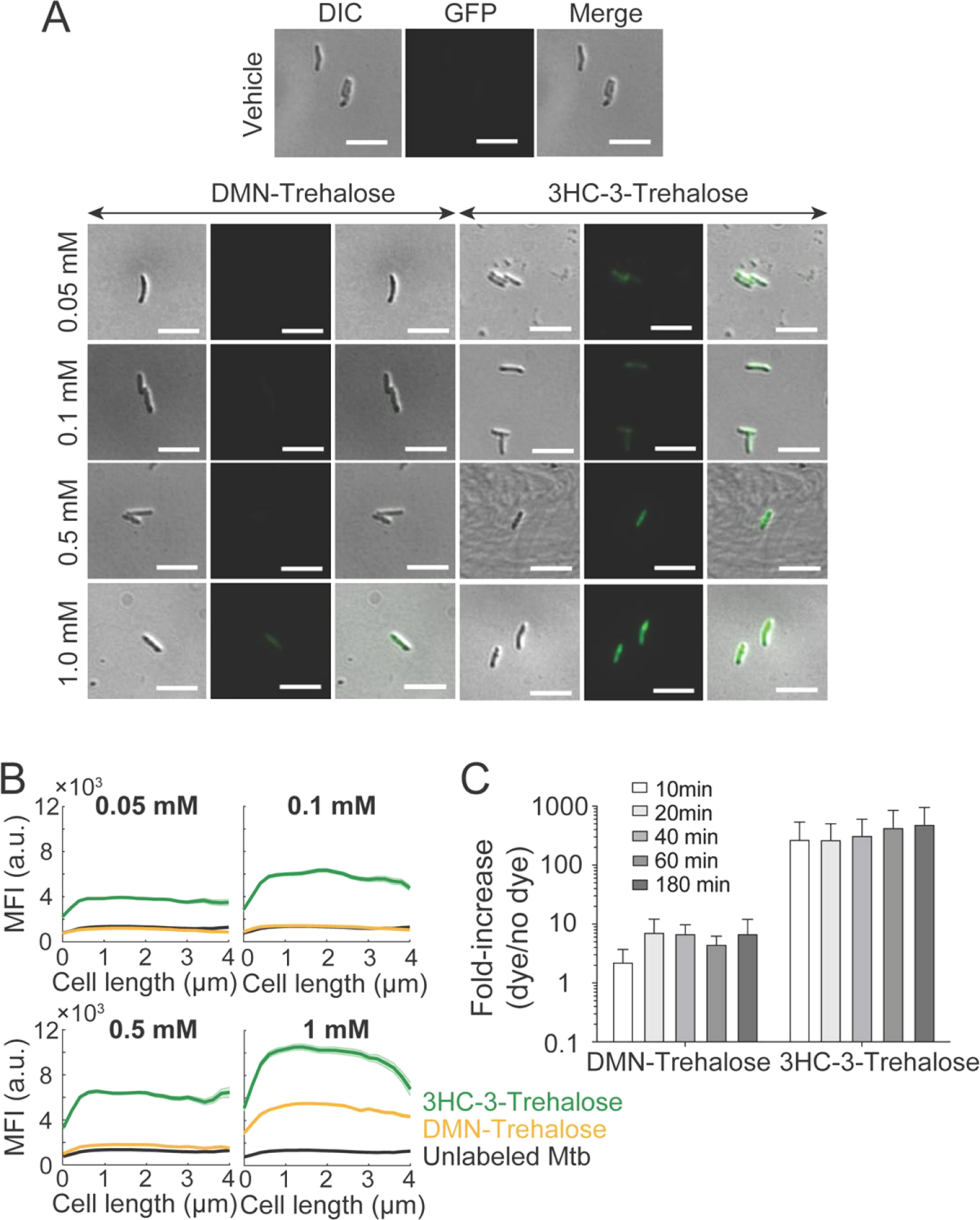
Mtb cells labeled with 3HC-3-Tre exhibit increased fluorescence intensities and can be detected within 10 min by flow cytometry. (A,B) Log-phase *Mtb* RvS cells (OD_600nm_ ∼ 0.5) were stained with various concentrations of DMN-Tre or 3HC-3-Tre (0.05, 0.1, 0.5, and 1 mM) for 3 h at 37 °C. The vehicle sample served as the unlabeled control. Mtb cells were then fixed in 2.5% glutaraldehyde for 1.5 h at 37 °C, washed twice with 1X PBS, and re-suspended in 100 μL 1X PBS. Ten microliters of each sample were spotted onto a 2% agarose pad and imaged with an epifluorescence microscope. Images are representative of each staining condition; scale bar represents 5 μm. Cells were then subjected to (A) microscopy analysis and (B) quantitative cell staining analysis (*n* = 85 cells for each condition). (C) Flow cytometry analysis of H37Rv Mtb cells labeled with 100 μM DMN-Tre or 3HC-3-Tre for 10, 20, 40, 60, and 180 min. Log-phase cultures (OD_600 nm_ ∼ 0.5) were stained with trehalose probes for the indicated times and then cell fluorescence was measured by flow cytometry. The fluorescence fold-increase (dye/nodye) was calculated as follows: fold-increase (dye/nodye) = mean fluorescence intensity of stained cells over the unlabeled sample. Data are means ± SEM from three independent biological experiments. Data were analyzed by two-way ANOVA test (ns: not significant).

Finally, we assessed whether 3HC-3-Tre probes permit rapid detection of stained Mtb cells (Figure 8, Figure S3). We first sought to determine the minimum concentration of probe required to detect labeled Mtb cells. We had previously shown that a concentration of 1 mM DMN-Tre was optimal for staining of axenic cultures of Mtb H37Rv.^19^ We hypothesized that the increased brightness of 3HC-3-Tre may permit detection at lower concentrations. We incubated axenic H37Rv Mtb cultures with 0, 0.05, 0.1, 0.5, and 1 mM DMN-Tre or 3HC-3-Tre for 3 h at 37 °C. Following a fixation step with glutaraldehyde, cells were washed, and 10 μL were smeared on 2% agarose pads for microscopy analysis. Consistent with our previous findings, Mtb cells stained with DMN-Tre concentrations ≤1 mM were not detectable using fluorescence (Figure 8A,B). At 1 mM DMN-Tre, cells were distinguishable from the unlabeled control, although the intensity remained lower than 3HC-3-Tre (Figure 8B). By contrast, Mtb cells stained with the lowest concentration of 3HC-3-Tre (0.05 mM) were readily detectable by microscopy and increasing concentrations of 3HC-3-Tre were associated with increased probe signal intensity (Figure 8B).

Finally, we sought to identify the minimum incubation time required for detection of 3HC-3-Tre-labeled Mtb. We incubated Mtb H37Rv cells with 100 μM DMN-Tre or 100 μM 3HC-3-Tre for 10, 20, 40, 60, and 180 min and then performed flow cytometry analysis (Figure 8C). Over the time course, the enhancement of DMN-Tre fluorescence in labeled versus unlabeled cells was consistenly <10-fold. By contrast, 3HC-3-Tre-labeled cells exhibited a >100-fold increase in fluorescence after 10 min, the earliest time point we measured. These results demonstrate the superior performance of the brighter 3HC-3-Tre probe for Mtb detection, which may ultimately prove best suited for point-of-care diagnosis.

## Discussion

Tuberculosis (TB) remains a major global health threat.^1^ As it stands, poor detection methods have contributed to millions of missed TB diagnoses in high-burden, endemic countries.^1,2^ Because of the lack of accurate detection at the point-of-care, TB transmission rates have been sustained in low-income countries. In this study, we report on a solvatochromic dye trehalose conjugate that can swiftly detect mycobacteria. 3HC-3-Tre is a robust fluorogenic probe that specifically labels Actinobacteria such as Mtb within 10 min and can be imaged without any wash steps. In addition to its utility for research, this set of attributes may enable the rapid detection of Mtb in sputum samples in low-resource settings.

Interestingly, despite 3HC probes’ high quantum fluorescence yields, we observed comparable fluorescence intensities when we subjected 3HC-3-Tre and DMN-Tre to various ratios of dioxane and water and analyzed fluorescence using a fluorimeter. By contrast, we observed 10-fold higher fluorescence intensities for 3HC-3-Tre-labeled Msmeg cells compared to DMN-Tre-labeled cells. There could be many reasons for this discrepancy. First, the disconnect could be related to intrinsic properties of the fluorophores in the conditions tested. Second, it could be differences in uptake efficiency of the two probes by Msmeg cells, which yielded increased fluorescence intensities for 3HC-3-Tre-labeled cells. This question is an area that will require further investigation by examining cell permeability more closely in follow-up studies.

Additionally, the probe-washout dynamics showed that Msmeg cells labeled with 3HC-3-Tre or 3HC-2-Tre exhibited a rate of fluorescence loss that was 2.3- and 5.9-fold higher respectively compared to DMN-Tre respectively, suggest additional labeling mechanisms beyond trehalose synthesis. The washout time scale for 3HC-2-Tre was much more rapid, reflecting trehalose-independent transient binding, and/or turn-on. By contrast, we confirmed that 3HC-3 and 3HC-3-Glc do not label Msmeg cells. Taken together, the data suggest 3HC-3-Tre fluorescence is likely induced following trehalose-dependent pathways and nonspecific/transient probe turn-on. Further studies are warranted to dissect in greater detail.

In future work, it may be possible to develop further red-shifted probes such as Nile-red dyes to minimize background fluorescence and maximize signal-to-background ratio, while maintaining minimal perturbations to mycobacteria. Furthermore, it is conceivable to easily synthesize trehalose moieties conjugated to an assortment of colors that span the fluorescence wavelength range to achieve multimodal labeling of mycobacteria. In addition to trehalose, the dyes could be coupled with other types of bacteria-specific molecules (sugars or amino acids) that would permit visualization of a subject of interest in various biological systems.

Beyond the performance of detection methods, TB diagnoses in low-income environments depend on many factors, such as stability, accessibility, and affordability.^2^ Solvatochromic dyes are highly stable at room temperature even in temperate environments, can potentially be coupled with a portable florescence-detection device, and a reasonable estimate of cost would be ≤40 cents per test. Because of these unique attributes, it is conceivable to use these reagents as mobile biomarkers for TB screening in resource-poor, remote environments.

Additionally, metabolic staining of mycobacteria provides an attractive avenue for assessing the presence of live bacteria and, hence by proxy, a measure of response to TB treatment. However, handling live organisms in routine laboratories or peripheral clinics is challenging due to the biosafety risk, and this aspect would need to be carefully considered. Options to address this risk include development of a collection system that allows for mixing of media containing metabolic stains directly to sputum collection vials, after sputum has been collected. After an incubation step, the bacteria can be fixed (thus inactivated) and imaged. This approach would require opening the sputum collection vial only once after collection to add the fixing agent and would pose no substantive additional risk to healthcare workers and lab staff. The development of probes that stain bacteria more efficiently, as reported here, would substantively increase the feasibility of this approach. Other alternatives include the use of collection vials that allow for mixing of fixing agents and stained live bacteria in a closed system. Thus, these novel probes can be used in service of both the scientific community to study mycobacterial metabolism, and the clinical community at large for TB detection in high-burden environments.

## Methods

### Fluorescence Spectra Procedures

One microliter of each dye-Tre conjugate (10 mM in water) was added to 1 mL of water or 99.9%, 99%, 95%, 90%, 75%, or 50% 1,4-dioxane in 1 cm × 0.4 cm quartz cuvettes (Starna Cells, Inc. 9F-G-10). Fluorescence data were acquired on a Photon Technology International Quanta Master 4 L-format scanning spectrofluorometer equipped with an LPS-220B 75-W xenon lamp and power supply, an A-1010B lamp housing with an integrated igniter, a switchable 814 photon-counting/analog photomultiplier detection unit, and an MD5020 motor driver. In the associated FelixGX v. 4.3.4.2010.6904 software, spectra were acquired using standard emission scan settings with the exception of the lamp slit widths, which were all set to 1 nm. Compounds were excited at 405, 488, 532, or 561 nm and emission intensity was monitored over 415–600 nm, 500–700 nm, 545–700 nm, or 575–750 nm, respectively. Data were exported as a text file and processed in Excel. Prism 7 (GraphPad) was used to create figures from the final data.

### General Procedures for Bacterial Culture Inoculation

Cultures of *Mycobacterium smegmatis* (Msmeg), *Corynebacterium glutamicum* (Cg), *Bacillus subtilis* (Bs), *Escherichia coli* (Ec), and *Staphylococcus aureus* (Sa) were grown as described previously.^6^ Briefly, Msmeg single colonies were inoculated in BD Difco Middlebrook 7H9 media (supplemented with 10% (v/v) oleate-albumin-dextrose-catalase (OADC), 0.5% (v/v) glycerol, and 0.5% (w/v) Tween 80) and incubated at 37 °C overnight. For Cg, Bs, Ec, and Sa, single colonies were inoculated in LB medium. Cg cultures were incubated at 30 °C, and Bs, Ec, and Sa cultures were incubated at 37 °C, all overnight.

### Metabolic Labeling Experiments

Experiments were performed as previously described.^6^ Briefly, overnight bacterial cultures were grown or diluted to an OD_600_ of 0.5 and aliquoted into Eppendorf tubes. The appropriate amount of stock dye (1 or 10 mM) was added to the aliquots to reach the indicated final concentration. Control samples were treated identically without the addition of any probes. Cultures were incubated for 1 h at 37 °C (Msmeg, Bs, Ec, Sa) or 30 °C (Cg). At the end of the experiment, samples were analyzed by microscopy and/or flow cytometry as described below.

### Confocal Microscopy

For no-wash imaging, a drop of sample (∼5 μL) was taken directly from the labeled culture. For wash imaging, cells were pelleted at 3300×*g* for 3 min, washed twice with 1X phosphate buffered saline (PBS) supplemented with 5% Tween80 (v/v), and resuspended in 300 μL 1X PBS. Subsequently, a drop of sample (∼5 μL) was spotted onto a 1% agarose pad on a microscope slide, allowed to dry, covered with a coverslip, and sealed with nail polish. Microscopy was performed on a Nikon A1R confocal microscope equipped with a Plan Fluor 60X oil immersion (NA: 1.30) objective. Samples were excited with a 405 nm violet laser, 488 nm blue laser, or 561 nm green laser, and imaged in the Aqua Amine (425–475 nm), FITC/GFP (500–550 nm), or RFP (570–620 nm) channels, respectively. NIS-Elements AR software (Nikon Inc.) and FIJI (ImageJ) v. 72 were used to process images. All image acquisition and processing were executed under identical conditions for control and test samples.

### Flow Cytometry

Cells were pelleted at 3300×*g* for 3 min, washed twice with 1X Dulbecco’s phosphate-buffered saline (DPBS; MT-21-030-CV, Thermo Fisher Scientific) supplemented with 5% Tween80 (v/v), and resuspended in 300 μL 1X DPBS. Fluorescence measurements were acquired in 5 mL culture tubes (14-959A, Thermo Fisher Scientific) suitable for flow cytometry. Experiments were performed on a BD LSR II.UV instrument in the shared Fluorescence Activated Cell Sorting (FACS) Facility at Stanford University. The instrument, excitation wavelengths, and filter sets used are noted in each figure or figure caption. Data were obtained for 10 000 cells per sample, processed using FlowJo, and imported into Prism 7 (Graphpad) for statistical analysis.

### Single-Cell Time-Lapse Microscopy

Single-cell time-lapse imaging was achieved using a microfluidic flow cell (CellASIC, B04A) and a custom temperature-controlled microscope system. Samples of mid log cultures (200 μL) were placed undiluted into loading wells. Wells containing 7H9 medium and 7H9 medium with dye were primed for 10 min at the target temperature under 5 psi. During the experiment, flow was set at 2 psi. Cells were imaged at 1 min time intervals using a Ti-Eclipse stand (Nikon Instruments) with a Plan Apo 100X DM Ph3 (NA: 1.45) (Nikon) objective and images were acquired with an iXon EM+ (Andor) camera. All cells were imaged in phase-contrast and TIRF illumination. Cells stained with dyes excited at peak GFP wavelength (488 nm; hydroxychromone dyes) were imaged under TIRF illumination to reduce background signal and photobleaching for which an OBIS laser (Coherent) light path was guided by an optical fiber to a TIRF illuminator (Nikon) and focused on the sample. Temperature was maintained at 37 °C using a stage-top incubator (Haison) coupled to a heater-controller (Air-Therm). Timing and control of the system was accomplished through μManager v. 1.41.^29^

### Image Analysis

Image stacks were imported into FIJI for initial data processing. Individual isolated cells were selected and cropped as image hyperstacks to include both phase-contrast and fluorescence channels. Phase-contrast images were used for automated segmentation analysis using Morphometrics^30^ in MATLAB (Mathworks), and outlines were overlaid on the corresponding fluorescence images for quantifying signal information. Further analyses were carried out using custom MATLAB scripts. Cellular intensity was normalized to the peak cellular intensity during labeling for dye constructs with high signal-to-background. The initial period of fluorescence intensity decay was quantified by nonlinear regression fitting to an exponential function (*I*(*t*) = *I*_0_ + *I*_1_*e*^–*γt*^). For dye constructs with no signal, cellular intensity was normalized by the mean signal-to-background of the cell to reflect the relative signal.

### Metabolic Labeling of Mtb

Mtb H37Rv or H37Ra cultures were grown via inoculation of a 1 mL frozen stock into 50 mL of Middlebrook 7H9 liquid medium supplemented with 10% (v/v) OADC enrichment (BBL Middlebrook OADC, 212351), 0.5% (v/v) glycerol, and 0.05% (w/v) Tween 80 (P1754, Sigma-Aldrich). Cells were grown to an OD_600_ of 0.5 to begin the experiments. Three biological replicates of 150 μL aliquots were incubated with DMN-Tre or 3HC-3-Tre for the indicated conditions. Time course: unlabeled (no dye), 10, 20, 40, 60, and 180 min (probe concentration 100 μM). Concentration dependence: cells were incubated with 0 (unlabeled control), 0.05, 0.1, 0.5, or 1 mM DMN-Tre or 3HC-3-Tre. Labeled cells were harvested by centrifugation (3000×*g* for 10 min) and then fixed in an equal volume of 4% paraformaldehyde or 2.5% glutaraldehyde (v/v). After the wash steps, cells were incubated at room temperature for at least 1 h with occasional rotation of the tube to ensure sterilization of all internal surfaces before microscopy and flow cytometry analysis.

### Statistical Analysis

Data are means ± SEM from at least two independent experiments. Unless otherwise specified, all data were analyzed using GraphPad Prism software’s analysis of variance (ANOVA) test, as specified in the figure legends.

## Supporting Information Available

Methods (including synthetic procedures), supporting schemes (Schemes S1 and S2), supplemental figures (Figures S1–S3), and supplemental tables. PDF)

## References

[ref1] World Health Organization. Global Tuberculosis Report 2019; World Health Organization S.l., 2019.

[ref2] World Health Organization. High-Priority Target Product Profiles for New Tuberculosis Diagnostics: Report of a Consensus Meeting; Geneva, Switzerland, 2014.

[ref3] Singhal R.; Myneedu V. P. Microscopy as a Diagnostic Tool in Pulmonary Tuberculosis. International Journal of Mycobacteriology 2015, 4 (1), 1–6. 10.1016/j.ijmyco.2014.12.006. 26655191

[ref4] Ziehl F. Zur Farbung Des Tuberkelbacillus. Dtsch. Med. Wochenschr. 1882, 8, 451 10.1055/s-0029-1196721.

[ref5] Ziehl F. Ueber Die Farbung Des Tuberkelbacillus. Dtsch. Med. Wochenschr. 1883, 9, 247–249. 10.1055/s-0029-1197158.

[ref6] Neelsen F. Ein Casuistischer Beitrag Zur Lehre von Der Tuberkulose. Centralblatt für die Medizinischen Wissenschaften 1883, 28, 497–501.

[ref7] Hagemann P. K. Fluoreszenzfärbung von Tuberkelbakterien Mit Auramin. Münchener Medizinische Wochenschrift 1938, 85, 1066–1068.

[ref8] Boyd J. C.; Marr J. J. Decreasing Reliability of Acid-Fast Smear Techniques for Detection of Tuberculosis. Ann. Intern. Med. 1975, 82 (4), 489–492. 10.7326/0003-4819-82-4-489. 1091188

[ref9] Truant J. P.; Brett W. A.; Thomas W. Fluorescence Microscopy of Tubercle Bacilli Stained with Auramine and Rhodamine. Henry Ford Hosp Med. Bull. 1962, 10, 287–296. 13922644

[ref10] Belisle J. T.; Vissa V. D.; Sievert T.; Takayama K.; Brennan P. J.; Besra G. S. Role of the Major Antigen of *Mycobacterium Tuberculosis* in Cell Wall Biogenesis. Science 1997, 276 (5317), 1420–1422. 10.1126/science.276.5317.1420. 9162010

[ref11] Swarts B. M.; Holsclaw C. M.; Jewett J. C.; Alber M.; Fox D. M.; Siegrist M. S.; Leary J. A.; Kalscheuer R.; Bertozzi C. R. Probing the Mycobacterial Trehalome with Bioorthogonal Chemistry. J. Am. Chem. Soc. 2012, 134 (39), 16123–16126. 10.1021/ja3062419. 22978752 PMC3466019

[ref12] Foley H. N.; Stewart J. A.; Kavunja H. W.; Rundell S. R.; Swarts B. M. Bioorthogonal Chemical Reporters for Selective In Situ Probing of Mycomembrane Components in Mycobacteria. Angew. Chem., Int. Ed. 2016, 55 (6), 2053–2057. 10.1002/anie.201509216. 26757001

[ref13] Backus K. M.; Boshoff H. I.; Barry C. S.; Boutureira O.; Patel M. K.; D’Hooge F.; Lee S. S.; Via L. E.; Tahlan K.; Barry C. E. III; Davis B. G. Uptake of Unnatural Trehalose Analogs as a Reporter for *Mycobacterium tuberculosis*. Nat. Chem. Biol. 2011, 7 (4), 228–235. 10.1038/nchembio.539. 21378984 PMC3157484

[ref14] Rundell S. R.; Wagar Z. L.; Meints L. M.; Olson C. D.; O’Neill M. K.; Piligian B. F.; Poston A. W.; Hood R. J.; Woodruff P. J.; Swarts B. M. Deoxyfluoro-D-Trehalose (FDTre) Analogues as Potential PET Probes for Imaging Mycobacterial Infection. Org. Biomol. Chem. 2016, 14 (36), 8598–8609. 10.1039/C6OB01734G. 27560008 PMC5026121

[ref15] Barry C. Exploiting the Biology of Trehalose to Develop Novel Imaging Probes for Tuberculosis. FASEB J. 2016, 30 (1_supplement), 503.1–503.1. 10.1096/fasebj.30.1_supplement.503.1.26527064

[ref16] Rodriguez-Rivera F. P.; Zhou X.; Theriot J. A.; Bertozzi C. R. Visualization of Mycobacterial Membrane Dynamics in Live Cells. J. Am. Chem. Soc. 2017, 139 (9), 3488–3495. 10.1021/jacs.6b12541. 28075574 PMC5345120

[ref17] Hodges H. L.; Brown R. A.; Crooks J. A.; Weibel D. B.; Kiessling L. L. Imaging Mycobacterial Growth and Division with a Fluorogenic Probe. Proc. Natl. Acad. Sci. U. S. A. 2018, 115 (20), 5271–5276. 10.1073/pnas.1720996115. 29703753 PMC5960302

[ref18] Cheng Y.; Xie J.; Lee K.-H.; Gaur R. L.; Song A.; Dai T.; Ren H.; Wu J.; Sun Z.; Banaei N.; Akin D.; Rao J. Rapid and Specific Labeling of Single Live *Mycobacterium tuberculosis* with a Dual-Targeting Fluorogenic Probe. Sci. Transl. Med. 2018, 10 (454), eaar4470 10.1126/scitranslmed.aar4470. 30111644 PMC6314683

[ref19] Kamariza M.; Shieh P.; Ealand C. S.; Peters J. S.; Chu B.; Rodriguez-Rivera F. P.; Sait M. R. B.; Treuren W. V.; Martinson N.; Kalscheuer R.; Kana B. D.; Bertozzi C. R. Rapid Detection of *Mycobacterium Tuberculosis* in Sputum with a Solvatochromic Trehalose Probe. Sci. Transl. Med. 2018, 10 (430), eaam6310 10.1126/scitranslmed.aam6310. 29491187 PMC5985656

[ref20] Klymchenko A. S.; Mely Y. Fluorescent Environment-Sensitive Dyes as Reporters of Biomolecular Interactions. Prog. Mol. Biol. Transl Sci. 2013, 113, 35–58. 10.1016/B978-0-12-386932-6.00002-8. 23244788

[ref21] Sahile H. A.; Rens C.; Shapira T.; Andersen R. J.; Av-Gay Y. DMN-Tre Labeling for Detection and High-Content Screening of Compounds against Intracellular Mycobacteria. ACS Omega 2020, 5 (7), 3661–3669. 10.1021/acsomega.9b04173. 32118181 PMC7045496

[ref22] McLean A. M.; Socher E.; Varnavski O.; Clark T. B.; Imperiali B.; Goodson T. Two-Photon Fluorescence Spectroscopy and Imaging of 4-Dimethylaminonaphthalimide Peptide and Protein Conjugates. J. Phys. Chem. B 2013, 117 (50), 15935–15942. 10.1021/jp407321g. 24245815 PMC3938489

[ref23] Klymchenko A. S.; Pivovarenko V. G.; Ozturk T.; Demchenko A. P. Modulation of the Solvent-Dependent Dual Emission in 3-Hydroxychromones by Substituents. New J. Chem. 2003, 27 (9), 1336–1343. 10.1039/b302965d.

[ref24] Kucherak O. A.; Richert L.; Mély Y.; Klymchenko A. S. Dipolar 3-Methoxychromones as Bright and Highly Solvatochromic Fluorescent Dyes. Phys. Chem. Chem. Phys. 2012, 14 (7), 2292–2300. 10.1039/c2cp23037b. 22237699

[ref25] Kucherak O. A.; Didier P.; Mély Y.; Klymchenko A. S. Fluorene Analogues of Prodan with Superior Fluorescence Brightness and Solvatochromism. J. Phys. Chem. Lett. 2010, 1 (3), 616–620. 10.1021/jz9003685.

[ref26] Klymchenko A. S.; Ozturk T.; Pivovarenko V. G.; Demchenko A. P. A 3-Hydroxychromone with Dramatically Improved Fluorescence Properties. Tetrahedron Lett. 2001, 42 (45), 7967–7970. 10.1016/S0040-4039(01)01723-3.

[ref27] Gunduz S.; Goren A. C.; Ozturk T. Facile Syntheses of 3-Hydroxyflavones. Org. Lett. 2012, 14 (6), 1576–1579. 10.1021/ol300310e. 22400900

[ref28] Junior C. O. R.; Castro S. B. R.; Pereira A. A.; Alves C. C. S.; Oliveira E. E.; Rêgo R. T.; Ferreira A. P.; de Almeida M. V. Synthesis of Genistein Coupled with Sugar Derivatives and Their Inhibitory Effect on Nitric Oxide Production in Macrophages. Eur. J. Med. Chem. 2014, 85, 615–620. 10.1016/j.ejmech.2014.08.032. 25127153

[ref29] Edelstein A.; Amodaj N.; Hoover K.; Vale R.; Stuurman N. Computer Control of Microscopes Using MManager. Current Protocols in Molecular Biology 2010, 92 (1), 14.20.1–14.20.17. 10.1002/0471142727.mb1420s92. PMC3065365 20890901

[ref30] Ursell T.; Lee T. K.; Shiomi D.; Shi H.; Tropini C.; Monds R. D.; Colavin A.; Billings G.; Bhaya-Grossman I.; Broxton M.; Huang B. E.; Niki H.; Huang K. C. Rapid, Precise Quantification of Bacterial Cellular Dimensions across a Genomic-Scale Knockout Library. BMC Biol. 2017, 15 (1), 17 10.1186/s12915-017-0348-8. 28222723 PMC5320674

